# Complete Genome Sequence of Thermus thermophilus Strain HB5018, Isolated from Mine Hot Spring in Japan

**DOI:** 10.1128/MRA.00039-21

**Published:** 2021-03-11

**Authors:** Kentaro Miyazaki, Toshiyuki Moriya, Naoki Nemoto, Tairo Oshima, Kei Yura, Yoshitaka Bessho

**Affiliations:** a Bioproduction Research Institute, National Institute of Advanced Industrial Science and Technology (AIST), Tsukuba, Ibaraki, Japan; b Department of Computational Biology and Medical Sciences, The University of Tokyo, Kashiwa, Chiba, Japan; c Institute of Environmental Microbiology, Kyowa Kako Co. Ltd., Machida, Japan; d Faculty of Advanced Engineering, Chiba Institute of Technology, Narashino, Chiba, Japan; e Graduate School of Humanities and Sciences, Ochanomizu University, Bunkyo, Tokyo, Japan; f Center for Interdisciplinary AI and Data Science, Ochanomizu University, Bunkyo, Tokyo, Japan; g School of Advanced Science and Engineering, Waseda University, Shinjuku, Tokyo, Japan; h RIKEN SPring-8 Center, Sayo, Hyogo, Japan; i Institute of Biological Chemistry, Academia Sinica, Taipei, Taiwan; Portland State University

## Abstract

We isolated Thermus thermophilus strain HB5018 from Mine Hot Spring in Japan, where the type strain HB8 was isolated nearly half a century ago. The complete genome sequence of HB5018 showed 99.1% average nucleotide identity with HB8, suggesting strict species conservation in the habitat over the past 50 years.

## ANNOUNCEMENT

An extreme thermophile, Thermus thermophilus, which grows optimally between 70°C and 75°C, was first isolated by Tairo Oshima about 50 years ago at Mine Hot Spring (34.7567N, 138.9828E) in Japan (1). Since then, many T. thermophilus strains have been isolated from high-temperature environments worldwide (2–7). Among them, strains HB8 (type strain) and HB27, both of which were isolated from Mine Hot Spring, have been extensively studied, particularly for proteins (8–13) and ribosomes (14–16), taking advantage of their high thermostability. Since 1968, we have repeatedly visited Mine Hot Spring and screened for thermophiles, but the vast majority of the isolates have been attributed to T. thermophilus. We have been interested in long-range species conservation in this unique habitat and initiated “fixed-point microbial community analysis” at the site.

In 2018, we collected a boiling water sample at Mine Hot Spring to screen for thermophiles. The sample was spread over *Thermus* medium (ATCC medium 697) agar plates (4.0% [wt/vol]) containing 0.4 mM MgCl_2_ and 0.35 mM CaCl_2_. After incubation at 70°C overnight, dozens of orange-yellow colonies appeared on the plates. We selected one of the strains, designated HB5018, for whole-genome sequence analysis. Cells were grown to saturation at 70°C in *Thermus* medium containing 0.4 mM MgCl_2_ and 0.35 mM CaCl_2_, and genomic DNA was purified using a blood and cell culture DNA midikit (Qiagen). Sequencing was performed by combining GridION (Oxford Nanopore Technologies [ONT]) and MiSeq (Illumina) technologies.

For long-read sequencing, unsheared genomic DNA (1 μg) was treated with a short-read eliminator kit (Circulomics), and a library was constructed using a ligation sequencing kit (ONT) and analyzed on a FLO-MIN106 R9.41 flow cell (ONT). For all software, default parameters were used except where otherwise noted. Base calling was conducted using Guppy v.4.0.11 to generate 43,824 reads (652 Mb) with an average length of 14,861.5 bases. The raw sequencing data were filtered (Q ≥ 10; read length, ≥1,000 bases) using NanoFilt v.2.3.0 (17), yielding 34,122 reads with a maximum read length of 303,772 bases, and an *N*_50_ value of 38,694 bases, spanning 558 Mb. For short-read sequencing, the Nextera DNA Flex library prep kit (Illumina) was used with the unsheared genomic DNA (500 ng) to generate libraries (∼700-bp inserts). Paired-end (2 × 256-base) sequencing was performed on a MiSeq instrument (Illumina), yielding 990,974 paired-end reads. Adapters and low-quality data were trimmed using fastp v.0.20.1 (18) (Q ≥ 30; read length ≥ 10 bases), yielding 628,050 paired-end reads with an average length of 217 bases, spanning 136 Mb.

The long- and short-read data were assembled *de novo* using Unicycler v.0.4.8 (19) and Flye v.2.8 (20) to yield structurally consistent assembled sequences, which were then iteratively polished with Pilon v.1.23 (21) to generate a single circular chromosome of 1,954,551 bp (GC content, 69.3%) and four circular plasmids (pHB5018b through pHB5018e). Rotation and circularity were confirmed via Unicycler (Table 1). Automatic annotation using DFAST v.1.2.4 (22) revealed that the chromosome contained 2,090 protein-coding, 50 tRNA, and 6 rRNA genes. A JSpecies analysis (23) revealed that the HB5018 chromosome showed the highest average nucleotide identity (99.11%; 89.12% aligned nucleotides) with that of HB8 (GenBank accession number NC_006461) among the known T. thermophilus genome sequences, suggesting strict species conservation in the habitat over 50 years.

**TABLE 1:** Genome statistics and features of Thermus thermophilus strain HB5018

Chromosome or plasmid	Length (bp)	GC content (%)	No. of CDSs^*a*^	No. of rRNAs	No. of tRNAs	GenBank accession no.
Chromosome	1,954,551	69.3	2,090	6	50	AP024270
Plasmid, pHB5018b	228,111	68.2	315	0	1	AP024271
Plasmid, pHB5018c	158,651	66.3	188	0	1	AP024272
Plasmid, pHB5018d	109,754	69.6	126	0	1	AP024273
Plasmid, pHB5018e	11,614	70.0	18	0	0	AP024274

a CDSs, coding DNA sequences.

### Data availability.

The complete genome sequence of T. thermophilus HB5018 is available from DDBJ/EMBL/GenBank under the accession numbers summarized in Table 1. The raw sequencing data were deposited in the SRA under the accession number DRA011331 (BioProject accession number PRJDB11006 and BioSample accession number SAMD00269939).

## References

[1] Oshima T, Imahori K. 1974. Description of *Thermus thermophilus* (Yoshida and Oshima) comb. nov., a nonsporulating thermophilic bacterium from a Japanese thermal spa. Int J Sys Evol Microbiol 24:102–112. doi:10.1099/00207713-24-1-102.

[2] Lyon P-F, Beffa T, Blanc M, Auling G, Aragno M. 2000. Isolation and characterization of highly thermophilic xylanolytic *Thermus thermophilus* strains from hot composts. Can J Microbiol 46:1029–1035. doi:10.1139/w00-075. 11109491

[3] Miyazaki K, Tomariguchi N. 2019. Complete genome sequences of *Thermus thermophilus* strains AA2-20 and AA2-29, isolated from Arima Onsen in Japan. Microbiol Resour Announc 8:e00820-19. doi:10.1128/MRA.00820-19. 31371550 PMC6675998

[4] Miyazaki K. 2019. Complete genome sequencing of *Thermus thermophilus* strain HC11, isolated from Mine Geyser in Japan. Microbiol Resour Announc 8:e00873-19. doi:10.1128/MRA.00873-19. 31558631 PMC6763646

[5] Fujino Y, Kawatsu R, Inagaki F, Umeda A, Yokoyama T, Okaue Y, Iwai S, Ogata S, Ohshima T, Doi K. 2008. *Thermus thermophilus* TMY isolated from silica scale taken from a geothermal power plant. J Appl Microbiol 104:70–78. doi:10.1111/j.1365-2672.2007.03528.x. 17850299

[6] Santos MA, Williams RAD, Da Costa MS. 1989. Numerical taxonomy of *Thermus* isolates from hot springs in Portugal. Syst Appl Microbiol 12:310–315. doi:10.1016/S0723-2020(89)80079-7.

[7] Manaia CM, Hoste B, Gutierrez MC, Gillis M, Ventosa A, Kersters K, Da Costa MS. 1995. Halotolerant *Thermus* strains from marine and terrestrial hot springs belong to *Thermus thermophilus* (ex Oshima and Imahori, 1974) nom. rev. emend. Syst Appl Microbiol 17:526–532. doi:10.1016/S0723-2020(11)80072-X.

[8] Miyazaki K, Yaoi T, Oshima T. 1994. Expression, purification, and substrate specificity of isocitrate dehydrogenase from *Thermus thermophilus* HB8. Eur J Biochem 221:899–903. doi:10.1111/j.1432-1033.1994.tb18805.x. 8181473

[9] Yaoi T, Miyazaki K, Oshima T, Komukai Y, Go M. 1996. Conversion of the coenzyme specificity of isocitrate dehydrogenase by module replacement. J Biochem 119:1014–1018. doi:10.1093/oxfordjournals.jbchem.a021316. 8797105

[10] Yokoyama S, Hirota H, Kigawa T, Yabuki T, Shirouzu M, Terada T, Ito Y, Matsuo Y, Kuroda Y, Nishimura Y, Kyogoku Y, Miki K, Masui R, Kuramitsu S. 2000. Structural genomics projects in Japan. Nat Struct Biol 7:943–945. doi:10.1038/80712. 11103994

[11] Iino H, Naitow H, Nakamura Y, Nakagawa N, Agari Y, Kanagawa M, Ebihara A, Shinkai A, Sugahara M, Miyano M, Kamiya N, Yokoyama S, Hirotsu K, Kuramitsu S. 2008. Crystallization screening test for the whole-cell project on *Thermus thermophilus* HB8. Acta Crystallogr Sect F Struct Biol Cryst Commun 64:487–491. doi:10.1107/S1744309108013572. PMC2496871 18540056

[12] Kim K, Okanishi H, Masui R, Harada A, Ueyama N, Kuramitsu S. 2012. Whole-cell proteome reference maps of an extreme thermophile, *Thermus thermophilus* HB8. Proteomics 12:3063–3068. doi:10.1002/pmic.201100375. 22887638

[13] Miyazaki K. 2005. A hyperthermophilic laccase from *Thermus thermophilus* HB27. Extremophiles 9:415–425. doi:10.1007/s00792-005-0458-z. 15999224

[14] Schluenzen F, Tocilj A, Zarivach R, Harms J, Gluehmann M, Janell D, Bashan A, Bartels H, Agmon I, Franceschi F, Yonath A. 2000. Structure of functionally activated small ribosomal subunit at 3.3 angstroms resolution. Cell 102:615–623. doi:10.1016/S0092-8674(00)00084-2. 11007480

[15] Brodersen DE, Clemons WM, Jr, Carter AP, Wimberly BT, Ramakrishnan V. 2002. Crystal structure of the 30 S ribosomal subunit from *Thermus thermophilus*: structure of the proteins and their interactions with 16 S RNA. J Mol Biol 316:725–768. doi:10.1006/jmbi.2001.5359. 11866529

[16] Miyazaki K, Tomariguchi N. 2019. Occurrence of randomly recombined functional 16S rRNA genes in *Thermus thermophilus* suggests genetic interoperability and promiscuity of bacterial 16S rRNAs. Sci Rep 9:11233. doi:10.1038/s41598-019-47807-z. 31375780 PMC6677816

[17] De Coster W, D'Hert S, Schultz DT, Cruts M, Van Broeckhoven C. 2018. NanoPack: visualizing and processing long-read sequencing data. Bioinformatics 34:2666–2669. doi:10.1093/bioinformatics/bty149. 29547981 PMC6061794

[18] Chen S, Zhou Y, Chen Y, Gu J. 2018. fastp: an ultra-fast all-in-one FASTQ preprocessor. Bioinformatics 34:i884–i890. doi:10.1093/bioinformatics/bty560. 30423086 PMC6129281

[19] Wick RR, Judd LM, Gorrie CL, Holt KE. 2017. Unicycler: resolving bacterial genome assemblies from short and long sequencing reads. PLoS Comput Biol 13:e1005595. doi:10.1371/journal.pcbi.1005595. 28594827 PMC5481147

[20] Kolmogorov M, Yuan J, Lin Y, Pevzner PA. 2019. Assembly of long, error-prone reads using repeat graphs. Nat Biotechnol 37:540–546. doi:10.1038/s41587-019-0072-8. 30936562

[21] Walker BJ, Abeel T, Shea T, Priest M, Abouelliel A, Sakthikumar S, Cuomo CA, Zeng Q, Wortman J, Young SK, Earl AM. 2014. Pilon: an integrated tool for comprehensive microbial variant detection and genome assembly improvement. PLoS One 9:e112963. doi:10.1371/journal.pone.0112963. 25409509 PMC4237348

[22] Tanizawa Y, Fujisawa T, Kaminuma E, Nakamura Y, Arita M. 2016. DFAST and DAGA: Web-based integrated genome annotation tools and resources. Biosci Microbiota Food Health 35:173–184. doi:10.12938/bmfh.16-003. 27867804 PMC5107635

[23] Richter M, Rosselló-Móra R, Oliver Glöckner F, Peplies J. 2016. JSpeciesWS: a Web server for prokaryotic species circumscription based on pairwise genome comparison. Bioinformatics 32:929–931. doi:10.1093/bioinformatics/btv681. 26576653 PMC5939971

